# Evaluating the factor structure, reliability and validity of the Copenhagen Burnout Inventory-Student Survey (CBI-SS) among faculty of arts students of Ekiti State University, Ado-Ekiti, Nigeria

**DOI:** 10.1038/s41598-024-61310-0

**Published:** 2024-05-07

**Authors:** Kehinde Sunday Oluwadiya, Omolara Kikelomo Owoeye, Adekunle Olatayo Adeoti

**Affiliations:** 1 Department of Surgery, University of Sierra Leone Teaching Hospitals Complex, Freetown, Sierra Leone; 2 Department of English and Literary Studies, Ekiti State University, Ado-Ekiti, Nigeria; 3 Department of Medicine, College of Medicine, Ekiti State University, Ado-Ekiti, Nigeria

**Keywords:** Burnout, Copenhagen burnout inventory, Nigeria, Student, Validity study, Psychology, Medical research

## Abstract

The Copenhagen burnout inventory-student survey (CBI-SS) has shown promising psychometric properties in diverse student populations. This study aims to investigate the psychometric properties of the Nigerian version of the CBI-SS. This was a cross-sectional study of 635 students from Ekiti State University, Ado-Ekiti, Nigeria. Confirmatory factor analysis (CFA) was utilized to assess the CBI-SS validity. The reliability score of the CBI-SS was 0.957, ranging from 0.862 to 0.914 for the subscales. Correlation coefficients among the four CBI-SS factors ranged from 0.507 to 0.713. The CFA indicated an adequate goodness-of-fit for the four-factor model of the CBI-SS with the sample data. However, Item 10 was removed due to unacceptably low Average Variance Extracted score. The four factors demonstrated a negative correlation with both General Academic Self-Efficacy Scale and Cumulative Grade Point Average. Furthermore, both self-reported burnout and perceived course stress showed associations with the CBI-SS, where lower levels of burnout corresponded with lower median scores on the CBI-SS scales. This study underscores the significance of the CBI-SS in evaluating student burnout within our student population. The findings indicate that the CBI-SS is a highly reliable and valid instrument for assessing student burnout, suggesting its potential for effective utilization in the Nigerian academic context.

## Introduction

Burnout as a concept dates back to 1974 when Herbert Freudenberger described it as “feelings of failure and being worn out or wrung out, resulting from an overload of claims on energy, personal resources, or the spiritual strength of the worker”^1^. Over the subsequent years, various scholars have contributed to its definition and understanding^2^. Maslach, notably, introduced the best-known definition of burnout as a three-dimensional syndrome characterized by emotional exhaustion, depersonalization, and reduced personal accomplishment^3^. Cherniss further explored burnout as a process, detailing its progressive nature^4^. Additionally, Schaufeli and Enzmann offered a definition in 1997, viewing burnout as a gap between expectations, intentions, efforts, and the harsh realities of life^5^. In 2019, the World Health Organization recognized burnout as a syndrome resulting from chronic workplace stress that has not been successfully managed. It characterized burnout by three dimensions: feelings of energy depletion or exhaustion; increased mental distance from one's job, or feelings of negativism or cynicism related to one's job; and reduced professional efficacy^6^.

These varied approaches to defining burnout are also reflected in the psychometric instruments developed for measuring the phenomenon. The Maslach Burnout Inventory (MBI) has been reported as the predominant tool in the vast majority of burnout literature^7,8^. However, several methodological and conceptual issues have been noted with the inventory, prompting the development of other instruments, including the Copenhagen Burnout Inventory (CBI)^7^. In a systematic review comparing CBI and MBI, findings revealed the CBI either matches or is more sensitive than the MBI in determining the burden of burnout in health workers and students. The review noted a marginal increase in the types and domains captured by the CBI when compared to the MBI^9^. The CBI is becoming more popular worldwide than the MBI, according to the report. Studies outside of America and Europe are increasingly reporting the CBI, showing its global application and relevance. Compared to MBI studies, CBI studies had higher response rates, and this may be due to the CBI's simpler question style, which respondents find easier to comprehend and answer, improving participation and data quality^9^. Furthermore, the systematic review by Shoman et al., which focused on the psychometric properties of five widely used occupational burnout instruments—namely, the Maslach Burnout Inventory (MBI), Pines' Burnout Measure (BM), Psychologist Burnout Inventory (PBI), Oldenburg Burnout Inventory (OLBI), and Copenhagen Burnout Inventory (CBI)—found that only the CBI and OLBI met the necessary criteria for construct validity. ^10^

The CBI is a 19-item questionnaire measuring three dimensions of burnout^7,11,12^. The Personal Burnout scale includes six items measuring the degree of physical and psychological fatigue experienced by a person regardless of their participation in the workforce, thus serving as a generic burnout scale. The Work-related Burnout scale comprises seven items measuring the degree of physical and psychological fatigue related to work. Lastly, the Client-related Burnout scale, with six items, measures the degree of physical and psychological fatigue experienced by people who work with clients. The authors argued that the instrument's validity could be adapted for different professions^7^.

While the original CBI has been validated for use among various populations^13,14^, some researchers felt it needed further refinement to meet the specific needs of students^15^. Consequently, in 2013, Campos et al. developed the student version of the CBI by dividing the client-related burnout dimension into two: Colleagues-related Burnout and Teacher-Related Burnout^11^. Campos et al. emphasized the significance of both peer and teacher relationships as determinants of the burnout syndrome for students, stating, "It is important to highlight that, in our proposal (CBI-S), the questions regarding the third dimension were duplicated given that, for students, both relationships with peers and teachers can act as determinants of the burnout syndrome"^11^. These four scales have shown good reliability and criterion-related validity in various international studies^16–18^.

Despite the growing body of research on burnout, it remains understudied in Nigeria and, indeed, across Africa^8,19,20^. To our knowledge, no study has reported on the psychometric properties of any student-focused burnout inventory in Nigeria. Considering the high population of students in Nigeria and the significant health and academic implications of unexplored burnout among this large proportion of the population, we believe it is critical to validate the CBI-SS in the Nigerian context.

The aim of this study is to explore the psychometric properties of the CBI-SS among the students of the Faculty of Arts, Ekiti State University, Ado-Ekiti, Nigeria. We hypothesize that our study sample data will conform to the CBI-SS's four-factor structure, encompassing Personal Burnout, Studies-Related Burnout, Fellow Students-Related Burnout, and Lecturers-Related Burnout.

## Methodology

### Study site and participants

The study was conducted at the Faculty of Arts, Ekiti State University, Ado-Ekiti, Nigeria between September 27 and December 9, 2023. This faculty comprises seven departments and a student population of 3928, of which 674 (17.1%) responded to the survey. Table 1 shows the characteristics of the respondents with the overall student population. The male-to-female ratio in the faculty is 1:1.6, as shown in Table 1.

**Table 1:** Distribution of students by gender across various classes and courses in the faculty.

Course	100L		200L		300L		400L		Total		
	M	F	M	F	M	F	M	F	M (%)	F (%)	
English	89	211	70	173	57	137	75	161	291 (29.9%)	682 (70.1%)	973
Linguistics	89	160	70	87	96	150	64	90	319 (39.6%)	487 (60.4%)	806
History	81	122	85	119	73	108	79	96	318 (41.7%)	445 (58.3%)	763
Philosophy	54	99	57	53	114	111	57	52	282 (47.2%)	315 (52.8%)	597
French	10	3	2	13	2	28	69	101	83 (36.4%)	145 (63.6%)	228
TMA	64	81	56	83	52	51	44	81	216 (42.2%)	296 (57.8%)	512
Religious Studies*	4		17		22		6		49		49
Total	387	680	340	545	394	607	388	587	1509 (38.4%)	2419 (61.6%)	3928

*Record of gender distribution was not available.

*TMA* Theatre and media arts.

The university operates on a two-semester system, and courses in the Faculty of Art span four years. Student performance is evaluated using a credit-based system, known as the cumulative grade point average (CGPA), with scores ranging from 0 to 5.0. For grading purposes, the CGPA is categorized as follows: < 1.0 (Fail), 1.0–1.49 (Pass), 1.50–2.39 (Third Class Honours), 2.5–3.49 (Second Class Honours (Lower Division), 3.5–4.49 (Second Class Honours (Upper Division), and 4.5 and above (First Class Honours).

### Study instruments

The study instrument consisted of three sections:

*Section One*: This section included socio-demographic and academic-related items such as age, sex, undergraduate course, level, financial challenges, ill-health, CGPA, thoughts about quitting the course, perceived course stress, and self-reported burnout.

*Section Two*: This section contains the Copenhagen Burnout Inventory Student Version (CBI-SS), adapted from the Campos et. al modification of the original CBI for use among students^11^. This version of the CBI consists of a total of twenty-five questions across four domains, as follows: study-related burnout (7 items), and six items each for personal burnout, colleague-related burnout, and teacher-related burnout.

We modified the questionnaire to ensure clarity and relevance for our respondents. For example, all instances of “colleagues” were replaced with “students” and “teachers” with “lecturers”, and factor 3 was renamed 'Fellow Student Related Burnout' (FSRB) from 'Colleague Related Burnout.' Finally, the questionnaire items were reworded to make them suitable for agreement-based Likert-type responses: Strongly Agree (5), Agree (4), Neutral (3) Disagree (2), Strongly Disagree (1). For example, item 1 was changed from "How often do you feel tired?" to "I often feel tired". These final modifications were based on a pilot study which showed that the students were more comfortable with this version than with the version used by Campos and Maroco^11^.

*Section Three*: This section included the General Academic Self-Efficacy (GASE) scale, a five-item self-report measure of academic self-efficacy on a five-point Likert scale, ranging from 1 (Strongly Disagree) to 5 (Strongly Agree). An example item is, “I know I can pass the exam if I put in enough work during the semester”^21,22^.

### Data collection

The questionnaire was administered through a Google Form and is entirely anonymous. However, a column was added for participants willing to provide their email for re-test purposes to facilitate test–retest reliability. The faculty has several WhatsApp groups for its students, one for each class/level within a course, e.g., four for Philosophy. Each WhatsApp group is managed by a class captain, acting as the admin.

The data collection period spanned ten weeks, from September 27 and December 9, 2023. Messages were sent several times a week by OKO, one of the authors who is a lecturer in the faculty to the class captains, encouraging them to remind their groups to participate in the survey. Additionally, lecturers occasionally addressed students in classes to promote participation, emphasizing its voluntary nature.

### Sample size

Following the recommendations for confirmatory factor analysis (CFA) of psychometric measures, which suggest between 10 and 20 samples per item, we estimated a sample size of 500 for this study. Considering the potential for higher attrition and non-completion rates typical of online studies, our target was set at a minimum of 600 participants^16,23,24^.

### Data analysis

The data downloaded from Google Form in Microsoft Excel format, were imported into IBM SPSS Version 25 for initial cleaning, in preparation for Confirmatory Factor Analysis (CFA) using Structural Equation Modeling (SEM) in JASP Version 0.18.1.0^25,26^.

The data were prepared for CFA by examining for missing data, outliers and unengaged responses^27–29^. Unengaged responses are those records in which respondents have filled the survey form without paying attention to the questions and answering them consistently with the same number^27,29^. Thirty-nine (5.8%) of the 674 respondents were either unengaged or had missing data greater > 60% and were excluded from further analysis, leaving 635 respondents for the final analysis. For those with less than 60% missing data, missing responses were replaced with the medians of their two nearest neighbors. Both the Cronbach's Alpha and the McDonald's Omega (ω), with a minimum acceptable value of 0.70, were employed to evaluate the reliability of the model and its constituent factors^30^. Despite its limitations, we used Cronbach's Alpha due to its widespread recognition and historical precedent in measuring reliability. It allowed for comparison with other studies. McDonald's Omega was employed due to its capacity to offer a more precise assessment of internal consistency when multidimensional constructs are present. By employing this dual approach, a thorough evaluation of reliability is achieved, as it merges the familiarity of Cronbach's Alpha with the more nuanced perspectives provided by McDonald's Omega ^31^. Normality of the items was checked using Kolmogorov–Smirnov procedure.

CFA was conducted to verify if the factor structure proposed by Campos et al. in Brazil presented an adequate fit for this study's sample^11^. Upon conducting the Kolmogorov–Smirnov test, it was determined that all 25 items in the dataset were not normally distributed; consequently, the Diagonally Weighted Least Squares (DWLS) method, coupled with robust error calculation, was employed as the estimator for the CFA to accommodate the ordinal nature and distribution characteristics of the data^32–34^. Model fit indices— χ2/df, Comparative Fit Index (CFI), Tucker-Lewis Index (TLI), Root Mean Square Error of Approximation (RMSEA), and Standardized Root Mean Square Residual (SRMR)—were used to determine the model's fit to our data^35^. The Heterotrait-Monotrait (HTMT) ratio assessed discriminant validity, while Average Variance Extracted (AVE) evaluated convergent validity. Adequate convergent and discriminant validity were indicated by AVE > 0.50 and HTMT < 0.90^35,36^. When the model did not fit, factor loadings, modification indices, and residuals were examined for model re-specification^28,29^. The instrument's concurrent validity was evaluated by correlating it with CGPA and GASE, as well as by testing its association with self-reported burnout and stress.

### Ethical approval

An institutional ethical approval was obtained from the Ekiti State University Teaching Hospital Institutional Review Board, which approved the study protocol. The online survey form contained a detailed introductory section providing information about the study’s purpose, procedures, potential risks and benefits, confidentiality, and the participants’ right to withdraw from the study at any time. All study participants gave informed consent and voluntarily agreed to participate in the research, and the study was conducted in accordance with the relevant guidelines and regulations.

## Result

The validation process of the CBI-SS involved a total of 635 undergraduates from the seven departments of the Faculty of Arts (FAT) at Ekiti State University, Ado, South-West Nigeria. The demographic characteristics of the respondents from the seven departments are detailed in Table 2. The ratio of male to female respondents is 1:1.99, indicating that the proportion of female students participating in the survey was significantly higher than that of male students when compared to the overall student population of the faculty (p=0.015).

**Table 2:** Academic and sociodemographic characteristics of the respondents.

Characteristic	No (%)
Course	
English	95 (15.0%)
Linguistics	131 (20.6%)
History	160 (25.2%)
Philosophy	94 (14.8%)
French	93 (14.6%)
Theatre and media arts	47 (7.4%)
Religious studies	15 (2.4%)
Level	
100L	157 (24.7%)
200L	114 (18.0%)
300L	142 (22.3%)
400L	222 (35.0%)
Median CGPA	3.650 (IQR: 3.230–4.023)
Gender	
Male	212 (33.4%)
Female	423 (66.6%)
Median age (Years)	21.0 (IQR: 20.0–23.0)

### Construct validity

The Confirmatory Factor Analysis, applied to our sample data, revealed an adequate goodness-of-fit for the four-factor CBI-SS (χ2/df=1.42), CFI=0.996, TLI =0.995, RMSEA=.026 [95%CI: 0.019-0.035], SRMR= 0.050. Apart from item 10 (in the SRB factor), all other item loadings were significant and high, ranging from .697 (Item 2) to .896 (Item 4) for Personal Burnout; .540 (Item 7) to .873 (Item 13) for Study-Related Burnout; .712 (Item 17) to .884 (Item 16) for Fellow Student-Related Burnout; and .726 (Item 23) to .837 (Item 21) for Lecturer-Related Burnout. However, the factor loading for Item 10 of the Student- Related Burnout (SRB) dimension was only 0.123, falling below the recommended threshold of 0.5 for acceptable factor loading. Additionally, the residual variance for this item was above 1.0, suggesting that a substantial portion of its variance is not explained by its associated factor. The SRB latent factor of this item exhibited an AVE of 0.455, below the recommended minimum of 0.5. As a result of these two observations, we removed item 10, improving the AVE of SRB factor to 0.524. This result indicated that the convergent validity of the model was enhanced by eliminating SRB item 10. The HTMT ratios for the model varied between 0.561 and 0.818, values that are lower than 0.90, indicating satisfactory discriminant validity among the factors. These findings suggest that each factor in the model is sufficiently distinct, supporting the theoretical framework of the study.

### Reliability

The internal consistency of the questionnaire was high, with McDonald's Omega (ω) value of 0.957. McDonald's Omega (ω) for PB, SRB, FSRB, and LRB were respectively 0.867, 0.862, 0.914 and 0.882. The overall Cronbach alpha was 0.949, with individual values of 0.872 (PB), 0.864 (SRB), 0.915 (CRB), and 0.884 (LRB).

### Correlation

**Figure 1:**
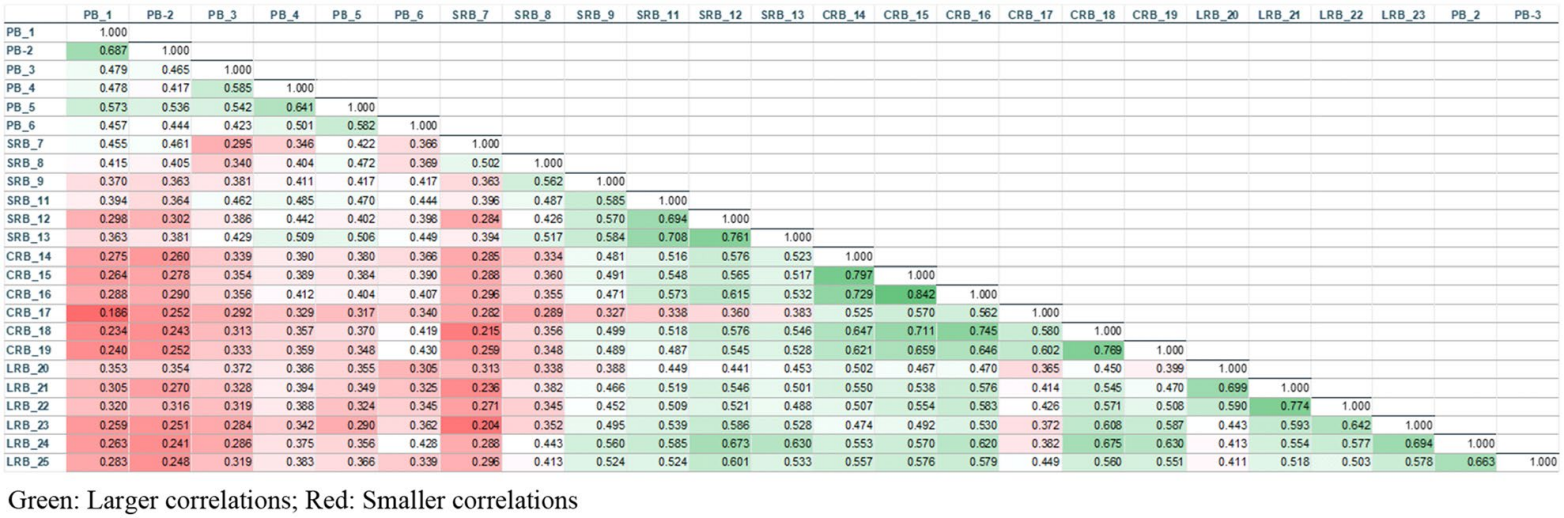
Inter-item correlation matrix of the CBI-SS.

As demonstrated in Table 3, the Spearman correlation coefficients among the four CBI-SS factors range from 0.507 (between LRB and PB) to 0.713 (between FSRB and LRB). This range suggests that these four factors are significantly related to constructs of burnout. Additionally, Figure 1 illustrates the correlation matrix for all 24 items of the CBI-SS. It shows that many of the within-subscale correlations among the items exhibit coefficient values exceeding 0.5, indicating a high inter-item correlation within the subscales. However, it is noteworthy that the cross-subscale correlations are generally lower compared to the within-scale correlations. This observation lends support to the factorial structure of the CBI-SS. The only item diverging from this trend of generally high subscale inter-item correlation (0.384–0.394) is Item SRB 7, which demonstrates low correlations with all its fellow subscale items except for Item 8. Additionally, this item records one of the lowest correlations (0.212–0.215) with other subscale items. This poor performance is mirrored in its confirmatory factor analysis (CFA) results, where it simultaneously exhibits the lowest factor loading (0.540) and the highest residual variance (0.779). These figures suggest that the variance in the item may be inadequately explained by its latent factor. However, we did not remove the item from the model because the model fit statistics were satisfactory. Moreover, some authorities advise against removing items from models unless absolutely necessary, as even poorly fitting items can contribute to the fit of other, more suitable items^29^.

**Table 3:** Inter-factor correlation of the CBS-SS.

	PB	SRB	FSRB	LRB
PB	1.000			
SRB	.663**	1.000		
FSRB	.527**	.637**	1.000	
LRB	.507**	.686**	.713**	1.000

### Criterion validity

The four factors demonstrate a negative correlation with both the General Academic Self-Efficacy scale and CGPA (Table 4). In addition, we examined the association between the CBI-SS subscales and self-reported burnout and perception of course as stressful using the Kruskal–Wallis test, a non-parametric method suitable for comparing medians across multiple groups. As Table 5 shows, students reporting definite burnout exhibited the highest median scores across all CBI-SS subscales (PB: 3.83, SRB: 3.67, FSRB: 3.17, LRB: 3.33), with IQRs indicating a range of responses within each category. A trend is observable where lower levels of self-reported burnout correspond with lower median scores in each subscale. This trend suggests a direct relationship between the degree of perceived burnout and the burnout subscales of the CBI-SS, for example, the category 'Not feeling burnout at all' consistently showed the lowest median scores for all subscales, which aligns with the expected outcomes of the CBI-SS in differentiating levels of burnout.

**Table 4:** Correlation between CBI-SS factors, GASE and CGPA.

	PB	SRB	FSRB	LRB
GASE	−.273**	−.387**	−.349**	−.417**
CGPA	−.131	−.148	−.154	−.205

**Significant at 0.001.

**Table 5:** Association between the CBI-SS subscales and self-reported burnout and perception of course as stressful.

	PB	SRB	FSRB	LRB
Are you currently feeling the effects of burnout?				
Definitely feeling burnout	3.83 (.92)	3.67 (1.0)	3.17 (1.0)	3.33 (1.0)
Somewhat feeling burnout	3.75 (1.0)	3.50 (1.0)	3.0 (1.17)	3.0 (1.21)
Not sure	3.33 (1.17)	3.0 (1.17)	3.0 (1.17)	3.0 (1.0)
Rarely feeling burnout	3.16 (1.33)	3.0 (1.08)	3.0 (1.0)	3.0 (1.08)
Not feeling burnout at all	3.0 (1.13)	2.5 (1.17)	2.67 (1.17)	2.5 (1.17)
I don’t know what burnout is	3.33 (1.13)	3.17 (1.71)	2.83 (1.38)	3.0 (1.25)
*P*-value	< 0.001	< 0.001	< 0.001	< 0.001
Do you consider this course stressful?				
Yes	3.67 (0.83)	3.50 (1.0)	3.17 (1.0)	3.17 (1.17)
No	3.08 (1.46)	2.83 (1.17)	2.92 (1.17)	3.0 (1.25)
Maybe	3.33 (1.0)	3.0 (1.17)	3.0 (1.0)	3.0 (1.0
*P*-value	< 0.001	< 0.001	< 0.001	< 0.001

The table also shows that students perceiving their course as ‘stressful’ recorded higher median scores on all subscales (PB: 3.67, SRB: 3.50, FSRB: 3.17, LRB: 3.17) compared to those not perceiving stress. The ‘Maybe’ response indicated uncertainty in course stress perception, yet this category also demonstrated elevated median scores, suggesting a potential link between uncertain stress perception and higher burnout scores.

## Discussion

This study explored the psychometric properties of the student version of the Copenhagen Burnout Inventory among students of the Faculty of Arts at the Ekiti State University, Ado-Ekiti. Significantly, this study represents the first comprehensive assessment of the reliability and validity of the CBI-SS within a Nigerian academic context, and indeed within the African continent as a whole. Additionally, it extends the validation of the CBI-SS beyond its original validation study in Brazil.

The Cronbach’s alpha coefficients of the CBI-SS were notably robust, ranging from 0.862 to 0.914 for all four subscales, with an overall value of 0.954 for the total scale. These reliability measures closely align with reported scores from the original Portuguese version of the CBI-SS (ranging from 0.875 to 0.931 for subscales and 0.957 for the total scale), as well as the Thai version (0.896 to 0.910 for all four subscales and 0.929 for the total scale)^11,16^. Moreover, both the Average Variance Extracted (AVE) and the Heterotrait-Monotrait (HTMT) ratios substantiate satisfactory levels of convergent and discriminant validity, respectively.

Significant and moderate to strong inter-factor correlations among the four CBI-SS dimensions indicate a considerable interdependence between the subscales. In light of this observation, prior scholars have proposed that an overall burnout score be derived from the four composite subscales of the CBI-SS^12,37^. Furthermore, an examination of the correlations between individual items in the four subscales revealed that they are moderately to strongly intercorrelated when compared to other items in the same subscale, but only weakly correlated when compared to items in other subscales. This observation provides significant support for the factorial design of the CBI-SS within our sample population.

The rationale behind Kristensen et al.’s development of the CBI as a replacement for the Maslach Burnout Inventory was, in part, that the MBI is a mismatch of three different components that should not be combined but studied in their own right^7^. They argued that depersonalization is a coping strategy for exhaustion while reduced personal accomplishment is a consequence of burnout. The high inter-subscale correlations and overall sound psychometric properties of the CBI-SS, as observed in our study, suggest it avoids this issue^11,37^. The fact that CBI-GS upholds the fundamental concept of burnout as energy depletion facilitates a clearer understanding of the causal relationship between burnout and other factors^7,37^.

Our study's results indicated that, similar to Campos et al.'s original adaptation of the CBI to a student population in Brazil, our data sample adequately fit the four-dimensional structure of the CBI-SS^11^. However, comparable adaptations in Portuguese and Brazilian contexts by Campos et al. and the Thai adaptation by Wongtrakul et al., achieving satisfactory fit necessitated the removal of one or more inventory items^11,16^. Specifically, in the Campos et al. adaptation, items 10 and 22 were excised due to low factor loading, resulting in only marginal fit improvements, as evidenced by the CFI and RMSEA values remaining below and above the respective thresholds. Similarly, the Thai adaptation involved the elimination of items 6, 10, and 17, along with the correlation of certain error terms during CFA. Our sample's fit surpassed these previous studies, maintaining adequate model fit parameters even with all items retained. However, the AVE for SRB and the factor loading for item 10 were below the accepted thresholds. When we removed item 10, the AVE for SRB increased above the 0.5 threshold. In the Thai version, items 4 and 7 were found to fit better in the SRB and Professional Burnout (PB) domains, respectively, rather than their original domains. This suggests that our model was more effective in fitting the sample than the two previous studies. We attribute this to two likely reasons. The first is that our study did not involve major translation, as Campos et al. provided an English version of their adaptation. The second reason could be our adaptation of the Likert-type options to ones more familiar to our students. Numerous authors have underscored the significance of language in the varying performances of psychometric measures across different countries and cultures^33–35^.

Similar to the findings of Campos et al. and Wongkuratul et al., the factor loading for Item 10 was lower than the recommended threshold^15,16^. This phenomenon has also been observed in studies on the general CBI among Chinese workers^38^. Previous researchers posited that this low loading might have occurred because Item 10 was the only one phrased negatively, leading them to speculate that respondents might not have realized the scale was reversed^11,37^. While this is a plausible explanation, we propose that the wording of the item itself ('I have enough energy for family and friends during leisure time') is problematic. Firstly, this item presupposes that students allocate time for leisure and intend to spend it with friends and family. However, as Olubor et al. noted, 'The Nigerian students have not embraced a culture of leisure in their lifestyle. Their primary concern is excelling in their studies'^39^. Nigerian students' limited leisure time is often underutilized due to various factors, including lack of time, anxiety, academic workload, and religious activities^39,40^. Furthermore, the advent of the internet and social media means that most students would prefer spending their leisure time online rather than with friends and family. Additionally, many of our students live far from their families, and the concept of 'friends' may be ambiguous since, for many, their closest friends are fellow students. This ambiguity might contribute to confusion about how to respond to this item. Yeh et al., who researched the original CBI, observed similar uncertainties among Chinese workers, noting that the item loaded on a work-related factor for men and on a general burnout factor for women^37^. Therefore, we recommend the removal of this item in future applications of the Nigerian version of the CBI-SS.

The CBI-SS demonstrated good concurrent and discriminant validity, as evidenced by its performance against some other measures of academic performance and school stress. Both the GASE and the CGPA were negatively correlated with the CBI-SS subscales. Furthermore, the CBI-SS showed a significant association with self-reported burnout and perceived course stress. The significant relationships between CBI-SS subscale medians and self-reported burnout levels highlight the impact of burnout on various facets of student life. A clear association between the CBI-SS subscales and both the perceived course stress and self-reported burnout emphasizes the importance of addressing stress and burnout among students. These results contribute valuable insights to the understanding of burnout in academic contexts and underline the need for effective support and policy initiatives to support student well-being.

## Conclusion

This study showcases the significance of the CBI-SS in evaluating student burnout within our student population. The study's findings demonstrate that the CBI-SS is a highly reliable and valid tool for evaluating student burnout, indicating its potential for effective assessment. The CBI-SS has shown strong Cronbach's alpha and McDonald's Omega (ω) coefficients and satisfactory levels of both convergent and discriminant validity in our sample. This indicates that it is a reliable and relevant tool for detecting burnout among students at Ekiti State University, Ado-Ekiti, Nigeria. The study's results emphasize the importance of considering cultural and contextual factors when using psychometric instruments, especially in terms of removing specific items to provide a good fit for the model. The strong correlation between the CBI-SS and measures of academic stress demonstrates the influence of burnout on students' lives. This study is a significant advancement in developing a more detailed and culturally aware understanding of academic burnout in the Nigerian student population. It also sets the stage for additional research and intervention in this essential element of student well-being and academic achievement.

### Limitation

As an online study whose participants were recruited via WhatsApp, this study is subject to the well-known limitations inherent to such methodologies, including the potential for selection bias^41^. Specifically, students who are more active on social media platforms or possess internet-enabled mobile devices may have been disproportionately likely to participate, potentially skewing the study's representativeness of the broader student population. Additionally, the study's cross-sectional design presents another limitation, as it constrains the ability to determine causal relationships from the data. Finally, we lacked access to the university's general student data, which would have allowed us to assess how representative our cohort was of the entire student body. However, these limitations notwithstanding, the study boasts the distinct advantage of having a large sample size. This factor is particularly valuable, as it offers insights into a poorly studied phenomenon within an under-explored environment.

## Data Availability

The datasets generated during and/or analysed during the study are available in the Open Science Framework (OSF) website at https://osf.io/5etxf/?view_only=f1e17cd1ee85409ca7dc34e436e9519b

## Abbreviation

OKO: Omolara Kikelomo Owoeye
